# Click Chemistry for Well-Defined Graft Copolymers

**DOI:** 10.3390/polym16233275

**Published:** 2024-11-25

**Authors:** Muhammad Faizan Ali, Bungo Ochiai

**Affiliations:** Department of Applied Chemistry, Chemical Engineering, and Biochemical Engineering, Graduate School of Science and Engineering, Yamagata University, 4-3-16 Jonan, Yonezawa 992-8510, Yamagata, Japan; t246815d@st.yamagata-u.ac.jp

**Keywords:** click chemistry, graft copolymers, copper-catalyzed azide-alkyne cycloaddition (CuAAC), thiol-ene click reactions, thiol-yne click reactions, grafting-from method, grafting-onto method, grafting-through method, controlled radical polymerization, polymer functionalization

## Abstract

Graft copolymers have gained significant importance in various fields due to their tunable functionality and well-defined architecture. However, there are still limitations due to the compatibility of monomers and functional groups depending on the polymerization mode. Click chemistry has solved this problem through its ability to easily and quantitatively link a wide range of polymers and functional groups. The combination of click chemistry, including copper-catalyzed azide-alkyne cycloaddition (CuAAC), thiol-ene, and thiol-yne reactions, with various polymerization techniques offers a promising solution for the robust and efficient preparation of graft copolymers with the desired architecture and functionality. In this review, we present successful applications of click chemistry in the production of well-defined graft copolymers with diverse functionalities such as for electronics, energy devices, biomedical applications, and nanotechnology.

## 1. Introduction

**Figure 1:**
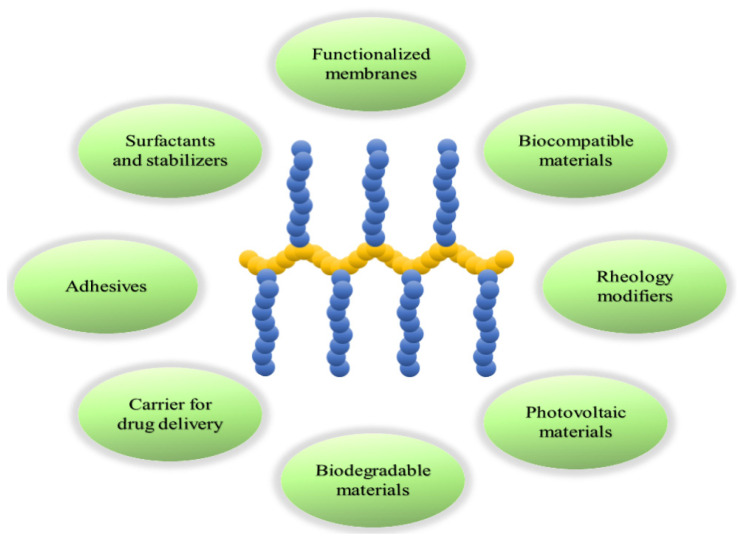
Applications of graft copolymers in various fields.

Graft copolymers are a type of copolymer where multiple polymer chains (graft or grafted chains) are attached to another polymer chain (backbone or stem chain) [1]. Both grafted and backbone chains tend to have extended conformations originating from the low freedom of conformation and steric repulsion of graft chains, which restrict the entanglement of the chains, resulting in higher solubility and lower viscosity of their solutions than analogous linear copolymers [2]. Backbone and grafted chains can be designed to attain specific properties, such as polarity, electronic and optical properties, and reactivity, and their appropriate cooperation provides properties that are unattainable with other polymeric architectures [3]. The mechanical properties and solubility are mainly governed by graft chains surrounding their backbone. This versatility in design and unique morphology make graft copolymers useful for a wide range of applications, including water-dispersible nano-cargos capable of transporting drugs and other biological substances [4,5,6], nanostructured materials [7], advanced surfactants [8,9,10,11], advanced photonic materials [12,13,14], and tough renewable materials [15] (Figure 1).

Preparations of graft copolymers often face the difficulty of suppressing side reactions and steric regulation by polymeric structures. Accordingly, high selectivity and efficiency are required for both polymerization and introduction of reactive groups. For polymerization, controlled radical polymerization (CRP) techniques, including nitroxide-mediated polymerization (NMP), atom transfer radical polymerization (ATRP), and reversible addition-fragmentation chain transfer (RAFT) polymerization, are attractive methodologies with facileness, a wide range of available monomers, and broad applicability to various polymerization conditions and functional groups [16,17,18,19,20]. Each method has its advantages and limitations, and available monomers and polymerization media depend on the polymerizations [21].

For reactions to connect reactive groups and polymers, click chemistry provides attractive solutions for producing complex macromolecular architectures [22,23,24]. Click chemistry was introduced in 2001 by Sharpless and coworkers and was awarded the Nobel Prize in Chemistry [25]. Click chemistry is a set of highly efficient chemical reactions that do not require harsh reaction conditions and tedious procedures. Additionally, these reactions are highly tolerant to impurities and sometimes can be operated in aqueous media. Furthermore, reactions of click chemistry are designed to avoid complex purification techniques. The features made click chemistry emerge as a green strategy for the design of myriads of functional molecules.

Cu(I)-catalyzed alkyne-azide cycloaddition (CuAAC) is the most widely used click reaction [26]. Alkyne groups couple with azide groups to give triazole rings under a wide range of conditions. Cu(I) catalysts enable quantitative reactions under ambient conditions. A new type of alkyne-azide cycloaddition was also investigated to avoid the use of metal catalysts due to the high demands from revolutionized biochemical research, where the toxicity of the metal catalysts negatively affects biological activities. The use of strained cyclooctynes enabled smooth bioorthogonal click reaction without metal catalysts, and this concept is called a strain-promoted alkyne–azide cycloaddition (SPAAC) reaction. The second Nobel Prize in Chemistry for click chemistry was awarded in 2022 for the development of click reactions and biorthogonal chemistry [27].

The addition of thiols to alkene and alkyne substrates is also a highly efficient method of click chemistry, referred to as thiol-ene and thiol-yne click reactions, respectively. These reactions are widely used in polymer chemistry due to their high efficiency, mild reaction conditions, and versatility in the synthesis of functional polymeric materials, resulting in the formation of new functional groups that can be used to fabricate a wide range of functional polymers with unique properties [28]. Radical addition in the presence of radical initiators and/or with UV irradiation is usually employed, although Michael-type nucleophilic addition can also be used for electron-deficient multiple bonds.

The combination of CRPs with click chemistry offers a powerful strategy for overcoming the limitations of the synthesis of graft copolymers and enhancing the versatility of structural designs. By leveraging the benefits of click chemistry, such as high efficiency, specificity, and compatibility with a variety of functional groups, the hybrid approach provides improved control over copolymer composition, structure, and properties. Additionally, the use of click chemistry can lead to the formation of unique structural motifs and architectures that are difficult to achieve with traditional CRP methods.

The range of click chemistry is expanding, and the other reactions involve Diels–Alder reactions and nucleophilic additions to strained rings. As a result, the area of applications of click chemistry has been expanding from drug designs to bioconjugation, designs of polymeric architectures, surface functionalization, etc.

This review summarizes the applications of click chemistry, mainly with CRPs, for the synthesis of graft copolymers. First, general synthetic procedures for graft copolymers are introduced with examples employing CRP without click reactions. Then, various syntheses of graft copolymers based on click couplings are reviewed by taking the synthetic aspects into account.

## 2. Synthetic Procedures for Graft Copolymers

**Figure 2:**
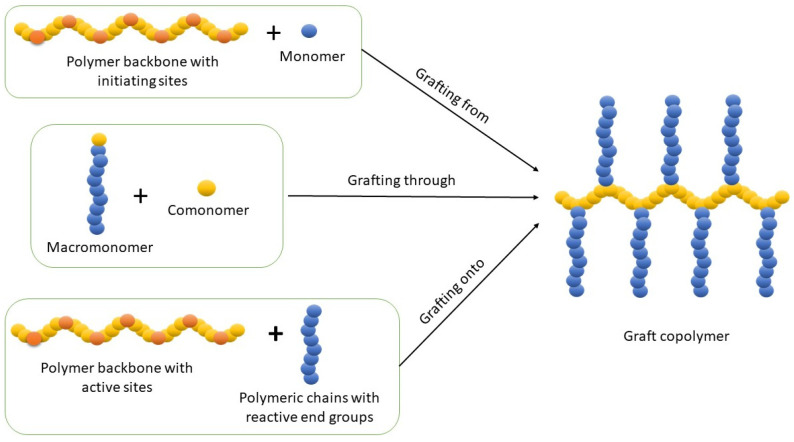
Procedures for synthesis of graft copolymers.

There are three basic procedures for the synthesis of graft copolymers: (i) grafting-from, (ii) grafting-onto, and (iii) grafting-through methods, in which monomers are polymerized from a polymer, polymers are connected to a polymer, and polymeric monomers are polymerized, respectively, to produce polymers with grafted chains (Figure 2). Each method is described with conventional non-click examples below.

### 2.1. Grafting-From Method

**Figure 3:**
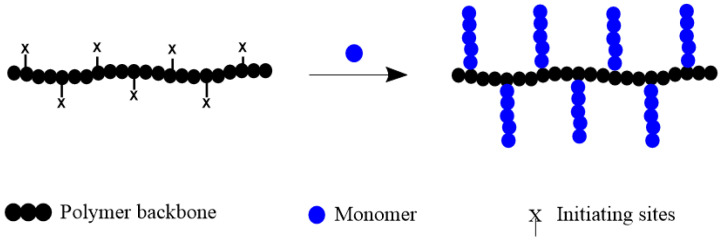
General scheme for polymerization of monomers onto polymerizable functionalities of polymeric backbone by grafting-from method.

In the grafting-from method, a polymeric backbone is first manufactured, and monomers are polymerized from initiating groups on the backbone, which are either inherently or subsequently introduced, resulting in the formation of grafted chains (Figure 3). The number of initiating groups introduced in the first chain defines the number of graft chains, and hence, the density of the graft chains can be tuned easily if the efficiency of initiation is quantitative. High grafting densities can be achieved due to localized initiation from stem polymer, but attaining uniform and predetermined lengths of grafts is challenging. Since typical initiated structures are chemically stable, the detailed characterization of each grafted segment by cleavage from the stem chain is basically difficult.

If the initiating structures are not inert to the polymerization for the preparation of backbones, subsequent introduction or protection–deprotection processes are necessary. For example, in grafting by radical polymerization from a polymeric backbone prepared by radical polymerization, the initiating groups are usually introduced by post-modification.

**Figure 4:**
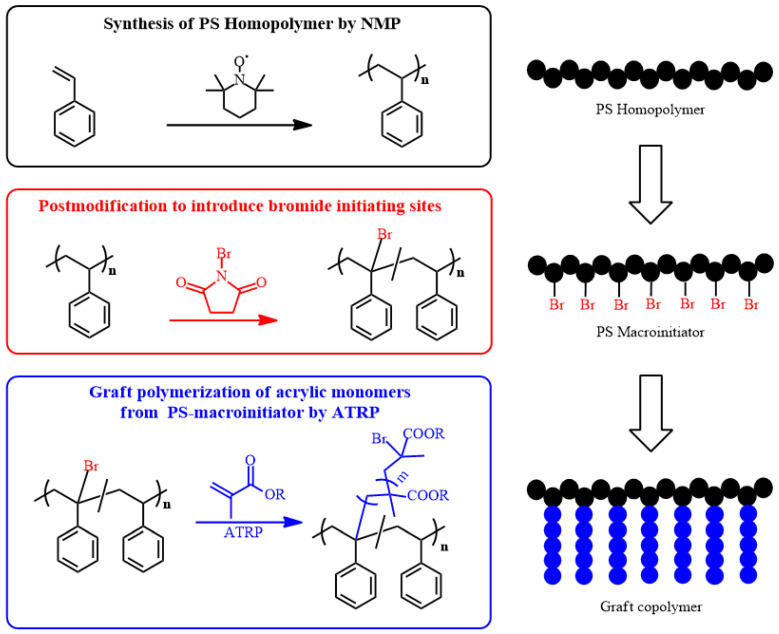
Synthesis of polystyrene precursor polymer by NMP, followed by post-modification and subsequent grafting-from ATRP of methacrylate monomers.

For instance, Vivek and coworkers graft-polymerized many types of monomers on polystyrene (PS) backbone by using the grafting-from method. The PS precursor polymer was synthesized by NMP at 130 °C using 2,2,6,6-tetramethyl-1-piperidinyloxy (TEMPO) as a mediator. The molecular weight was controlled as required by changing the polymerization time. Benzylic bromide moieties for the initiation were introduced to PS using *N*-bromosuccinimide (NBS). Various methacrylates and styrene were graft-copolymerized from this brominated PS by ATRP in the presence of CuBr and *N*,*N*,*N*′,*N*″,*N*″-pentamethyldiethylenetriamine (PMDETA) (Figure 4). Block copolymers can be prepared by subsequent polymerization of different monomers. For example, sequential polymerization of methyl methacrylate (MMA) after *t*-butyl acrylate (tBA) gave PS-*g*-(PMMA-*b*-PtBA) [29].

### 2.2. Grafting-Onto Method

**Figure 5:**
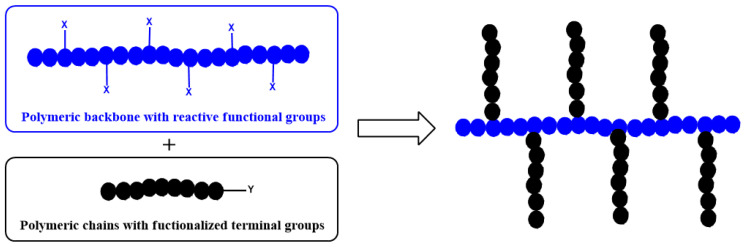
General scheme of producing graft copolymers by coupling of polymers with functional end group (Y) to reactive sites (X) of backbone polymer using the grafting-onto method.

The grafting-onto method comprises coupling polymers with one reactive chain end as graft chains to a backbone polymer bearing connecting groups (Figure 5). The backbone and grafting polymers are prepared separately using any polymerization technique. Accordingly, the polymers can be characterized before grafting, and their chain lengths and polydispersity may be precisely controlled by using living or controlled polymerizations. For stem chains, reactive functional groups for grafting can be introduced onto the backbones by (co)polymerization with a functional monomer, post-functionalization, or the formation of the functional group accompanied by polymerization. For graft chains, functional groups are introduced at one of the chain ends. For example, polymers with -SH termini can be synthesized by RAFT polymerization followed by cleavage of the RAFT termini at the living ends.

The molecular weight of the final graft copolymer can be defined by the molecular weight and number of connecting sites of the backbone polymer and molecular weights of grafts, if the coupling efficiency is high. This technique is advantageous for preparing graft copolymers with well-defined molecular weights, while it is disadvantageous for preparing densely grafted polymers due to the steric hindrance at the latter stage of the grafting reactions.

As an earlier attempt, Candau and coworkers reported a grafting-onto synthesis of amphiphilic PS-*g*-poly(ethylene oxide) in 1977 [30]. The PS backbone was synthesized by living anionic polymerization using *s*-butyllithium as an initiator in benzene. The active sites for grafting were incorporated by partial chloromethylation of benzene rings in PS with bis(chloromethyl) ether in the presence of SnCl_4_. The degree of chloromethylation was up to 11%.

**Figure 6:**
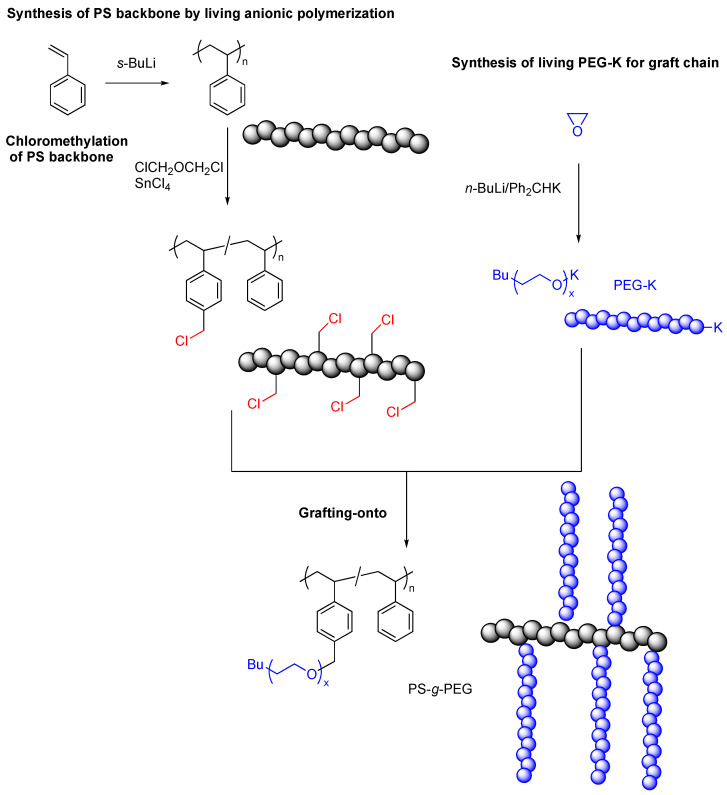
Grafting-onto synthesis of PS-*g*-PEG by grafting of living PEG-K onto chloromethylated PS.

Poly(ethylene glycol) (PEG) with a living monofunctional potassium alkoxide end (PEG-K) was separately prepared by anionic polymerization with an initiating system consisting of diphenylmethyl potassium and butyllithium. The grafting-onto reaction was carried out by adding chloromethylated PS into the solution of living PEG-K. The nucleophilic substitution of the benzylic chloride structures with living PEG-K resulted in the formation of well-defined PS-*g*-PEG (Figure 6). PS-*g*-PEG is amphiphilic and could be used as an emulsifier. Such designs have been applied for advanced applications like micro- and nano-emulsification by controlling the balance of polarity, chain lengths, and grafting densities.

### 2.3. Grafting-Through Method

**Figure 7:**
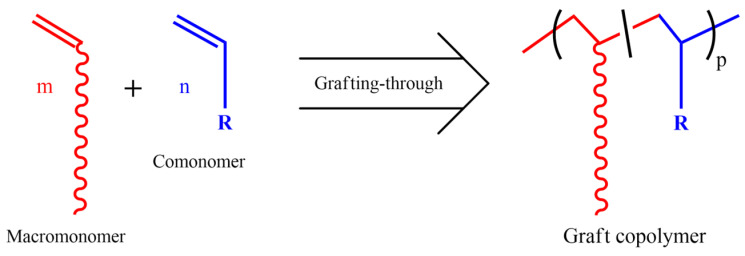
Grafting-through synthesis of graft copolymer by copolymerization of macromonomer.

The grafting-through method is a technique to produce graft copolymers by (co)polymerization of macromonomers. The structures, including the lengths of the graft chains, can be fully characterized before the grafting-through polymerization, while the polymerizable groups significantly smaller than the polymer chains often reduce the polymerizability. Figure 7 shows a simple scheme for polymerization of macromonomers to produce graft copolymers.

The polymerizable end group may be introduced by using initiating or terminating species bearing a polymerizable group or by post-modification. The use of the terminating agents and post-modification do not depend on the orthogonality of the polymerizable groups to the polymerization modes for the preparation of the macromonomers, while the quantitative introduction is sometimes difficult. Initiators with polymerizable groups are advantageous in the quantitative introduction, while it requires the inertness of the initiating species to the polymerization of the macromonomers and the absence of chain transfer.

**Figure 8:**
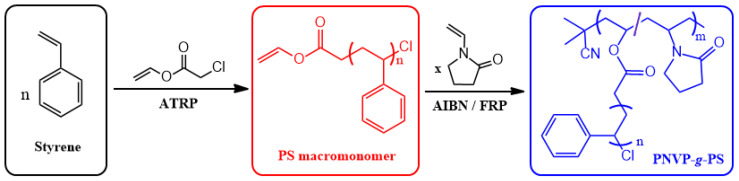
Synthesis of PNVP-*g*-PS by free radical grafting-through copolymerization of well-defined polystyrene macromonomer prepared by ATRP with *N*-vinyl pyrrolidinone.

The appropriate choice of initiating species and polymerization technique allows for facile preparation of macromonomers. For example, vinyl esters are effective, radically polymerizable end groups of macromonomers since they are inert to the conditions for ATRP of styrene, but can be copolymerized with appropriate vinyl monomers by the free radical mechanism. Matyjaszewski et al. applied this selectivity to grafting-through synthesis [31]. ATRP of styrene initiated with vinyl chloroacetate yielded well-defined PS macromonomers with a vinyl terminus (PSMMs) that was unreacted under the ATRP conditions. PSMMs were copolymerized with *N*-vinyl pyrrolidinone (NVP) by free radical polymerization (Figure 8) to produce a graft copolymer (PNVP-*g*-PS). Hydrophilic PNVP backbone and hydrophobic PS grafts produced an amphiphilic PNVP-*g*-PS structure that swelled in water and produced a hydrogel through the solvation of PNVP chains and physical cross-linkage of PS grafts. The contents of absorbed water reached up to 97% under equilibrium, indicating the potential application for absorbents.

The techniques mentioned above for producing graft copolymers—the grafting-from, grafting-onto, and grafting-through methods—possess their unique advantages and limitations. Grafting-from is ideal for achieving high degrees of grafting and tailoring the properties of the grafted chain, but it is often difficult to control the distribution and density of the grafted chains. Grafting-onto is advantageous for controlling the grafting density and distribution of grafted chains, but it involves difficulty in achieving a high degree of grafting. Grafting-through is suitable for achieving a high degree of grafting and obtaining block copolymers, but low reactivity of macromonomers often becomes a barrier to high conversion and molecular weight.

The choice of technique depends on the desired applications and properties of the resulting graft copolymer. A limitation of conventional grafting methods is the difficulty in quantitative and selective introduction of initiating or coupling sites on stem or graft polymers. The control often relies on knowledge based on accumulated experiments to tune the substitution degrees and retain reactive initiating sites.

## 3. Click Chemistry for Synthesis of Graft Copolymers

Synthesis of graft copolymers often suffers from side reactions of reactive groups and poor reactivity due to steric hindrance. Click chemistry has overcome this difficulty by virtue of its high reactivity and orthogonality to various functional groups. Among a variety of effective click chemistry types for graft copolymers, this review focuses on three major reactions, namely, CuAAC, thiol-ene, and thiol-yne reactions.

### 3.1. Cu-Catalyzed Azide-Alkyne Cycloaddition Click Reaction

**Figure 9:**
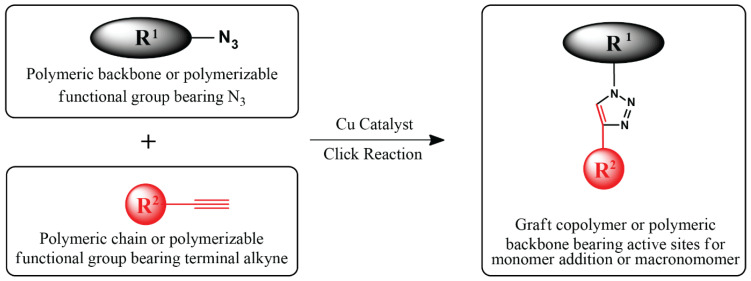
General scheme of CuAAC to produce graft copolymers.

CuAAC is the most widely used click reaction and is a powerful tool in fabricating graft copolymers. Either terminal alkyne or azide groups are installed in polymers or functional groups for coupling and polymerization, and their smooth coupling yields graft copolymers or precursors (Figure 9). Graft copolymers can be prepared either by grafting-from, grafting-onto, or grafting-through methods based on CuAAC.

For instance, a compound bearing both a yne-terminus and an initiating group can be coupled to a polymeric backbone bearing N_3_ pendant groups to produce a macro-initiator with multiple initiating sites. A graft copolymer may be produced by subsequent grafting-from polymerization of monomers.

**Figure 10:**
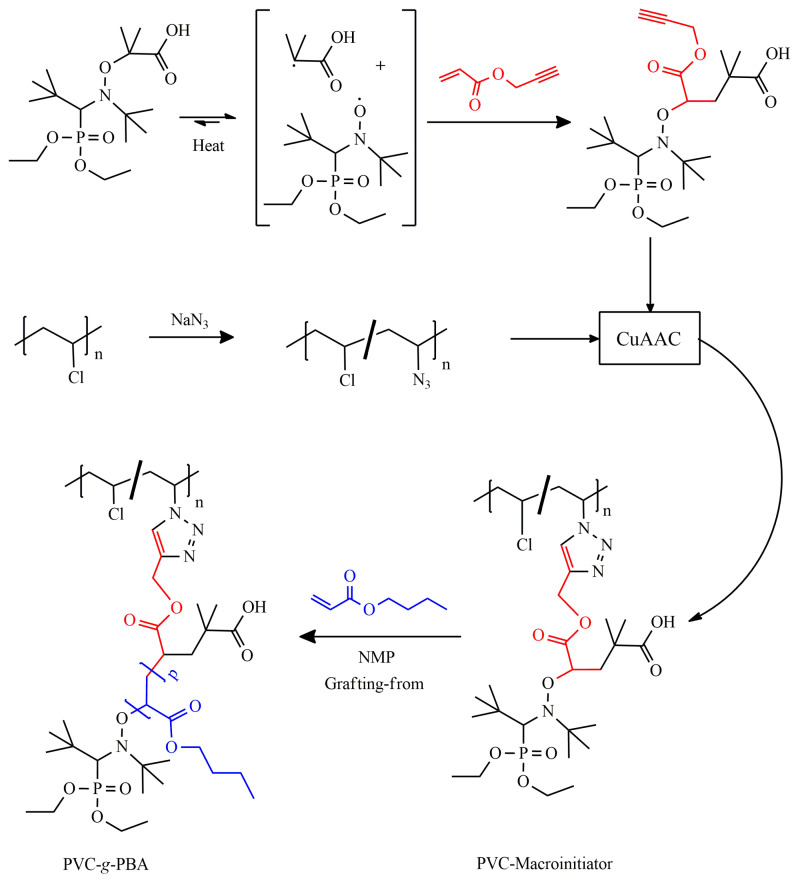
Synthesis of well-defined PVC-*g*-PBA by grafting-from polymerization of BA from PVC-macroinitiator containing nitroxide initiating sites produced by CuAAC click coupling of Alkoxyamine 2 and PVC-N_3._

Veber and coworkers developed a graft copolymer of poly(vinyl chloride) (PVC) and poly(*n*-butyl acrylate) (PBA) with tunable properties by controlled grafting-from polymerization of BA from PVC modified by a CuAAC click reaction (Figure 10) [20]. First, a nitroxide bearing a yne group (Alkoxyamine 2) was produced by intermolecular radical addition of propargyl acrylate and MAMA-SG1 (BlocBuilder MA) [32] under thermal activation. PVC-N_3_ was separately synthesized by reacting with NaN_3_, and the degree of substitution was controlled by regulating the temperature and reaction time.

A PVC-macroinitiator was prepared by CuAAC click introduction of nitroxide initiation sites through the reaction of Alkoxyamine 2 with PVC-N_3_ catalyzed by CuBr and 2,2’-bipyridine. Finally, the NMP of BA from the PVC-macroinitiator was carried out. The resulting PVC-*g*-PBA had a *T*_g_ value as low as −10.5 °C.

**Figure 11:**
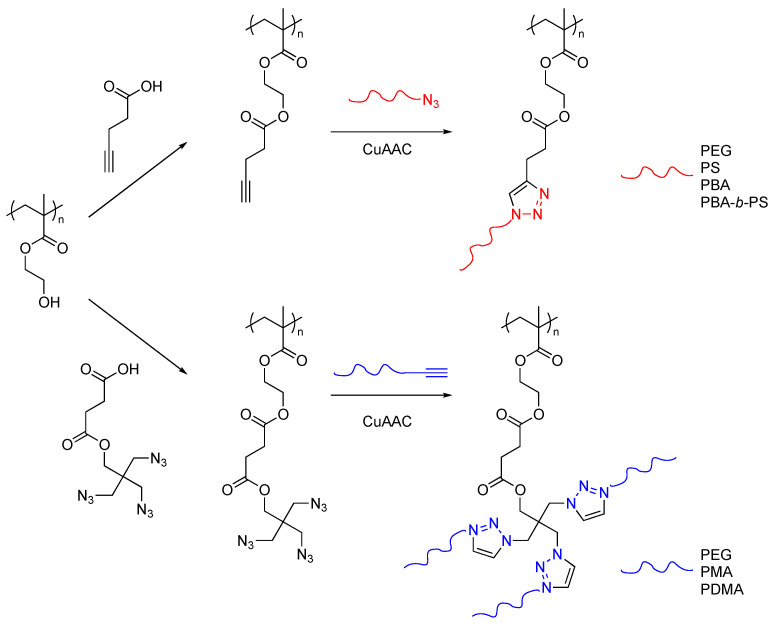
Synthesis of densely grafted copolymers by CuAAC grafting-onto method.

The high efficiency of CuAAC can solve the difficulty of the grafting-onto method in preparing dense graft copolymers. An earlier work on the synthesis of well-defined molecular brushes was reported by Matyjaszewski and Gao (Figure 11) [33]. Poly(hydroxyethyl methacrylate) (PHEMA) with controlled molecular weight was synthesized by ATRP and was reacted with pentynoic acid to yield a backbone polymer with alkynyl side chains. Five kinds of azide-termini polymers with different molecular weights were coupled with the multi-alkynyl polymer backbone, and various molecular brushes with different compositions and morphologies were produced. The grafting densities of vinyl polymers were less than 50% due to stearic hindrance, while that of thinner PEG reached 88.4% due to the lower stearic hindrance.

Gao et al. further extended this strategy to the synthesis of molecular brushes with ultra-high draft densities [34]. Polymethacrylate stem chains containing di- or tri-functional azide groups in the side chain were reacted with polymers bearing alkynyl terminus. The highest graft density was 1.34 side chains per backbone carbon atom; namely, the number of grafting chains exceeded the number of carbon atoms in the backbone. This excellent efficiency originated from the complexation of the triazole moieties with the Cu catalyst, assisting the introduction of grafting chains around already-installed grafted chains. As alkynyl termini polymers, PEG with different molecular weights, poly(methyl methacrylate), and poly(dimethyl acrylamide) prepared by RAFT polymerization using CTA bearing a terminal alkynyl group were employed.

**Figure 12:**
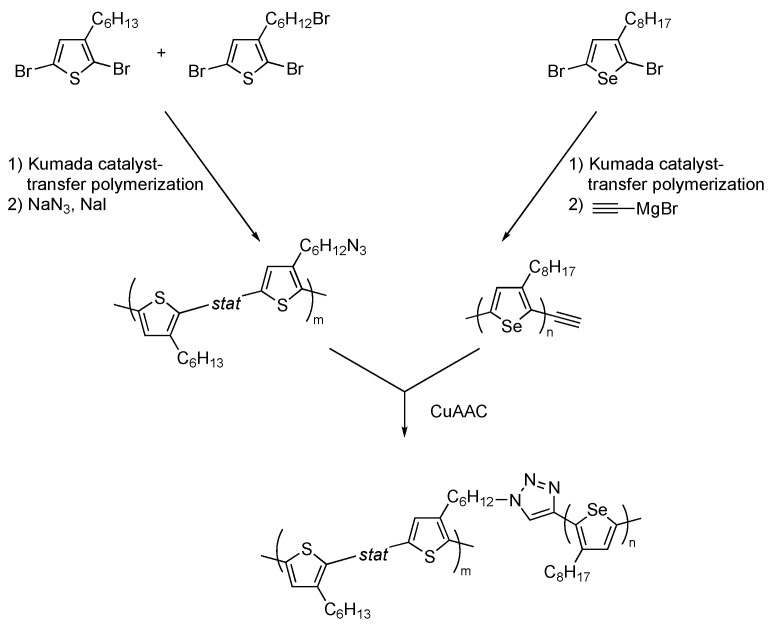
Graft-onto synthesis of polythiophene-*g*-polyselenophene by CuAAC.

The CuAAC grafting-onto strategy was also applied to the synthesis of conjugated graft copolymers potentially applicable to organic electronics. Obhi and Seferos et al. reported click-conjugation of azide-functionalized polythiophene backbones and alkyne-terminated polyselenophenes (Figure 12) [35]. Both the backbone and graft chains were prepared by controlled Kumada catalyst-transfer polymerization, and a series of polythiophene-*g*-polyselenophene with different grafting densities and lengths was prepared. Although the synthesis of graft copolymers with high grafting densities was challenging, midrange grafting density copolymers were fabricated by optimizing the precursor polymers and catalytic system.

Various end-functionalized polymers can be precursor macromonomers for graft copolymers, and mild conditions of CuAAC are suitable for introducing sensitive polymerizable groups. However, a drawback is the slower polymerization of macromonomers, preventing the complete consumption of macromonomers, which typically results in tedious purification. Vogt et al. demonstrated the applicability of CuAAC click coupling for synthesizing well-defined macromonomers and their subsequent polymerization to produce densely grafted copolymers [36].

**Figure 13:**
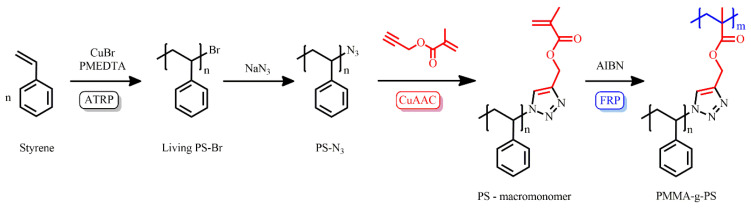
Synthesis of densely grafted copolymers by grafting through polymerization of PS-macromonomer, produced by CuAAC coupling of PS-N_3_ and propargyl methacrylate.

PS macromonomers were synthesized by sequential ATRP, end-group modification, and CuAAC introduction of the methacrylate group. ATRP of styrene using a CuBr/PMDETA catalyst gave living PS-Br, which was converted to PS-N_3_ by NaN_3_. A PS-macromonomer was prepared by CuAAC click coupling of PS-N_3_ with propargyl methacrylate using a CuBr/PMDETA catalyst (Figure 13). The radical polymerization of PS-macromonomer yielded densely grafted polymethylmethacrylate with densely grafted PS chains.

The combination of CuAAC and the grafting-through method can also be applied to the synthesis of all-conjugated graft copolymers. Poly(3-hexylthiophene) (P3HT) with an alkynyl end group was coupled with dibrominated donor-acceptor aromatic monomers bearing an azide group, and macromonomers were prepared [37]. Palladium-catalyzed Suzuki–Miyaura coupling polymerization with a carbazole monomer bearing two boronic acid ester groups yielded donor–acceptor polymers bearing P3HT as grafted chains.

### 3.2. Thiol-Ene Click Reaction

The hydrothiolation of alkenes, also known as thiol-ene chemistry, is also a frequently employed click reaction. Thiol-ene reactions proceed by both nucleophilic and radical attacks, but the radical process has been utilized more in the production of graft copolymers due to their applicability to a wide range of alkenes. Conventional approaches for thiols require a protection–deprotection process due to the reactivity of thiols in various reactions, but ring-opening additions and cleavages of RAFT terminating ends improved the accessibility towards thiols.

**Figure 14:**
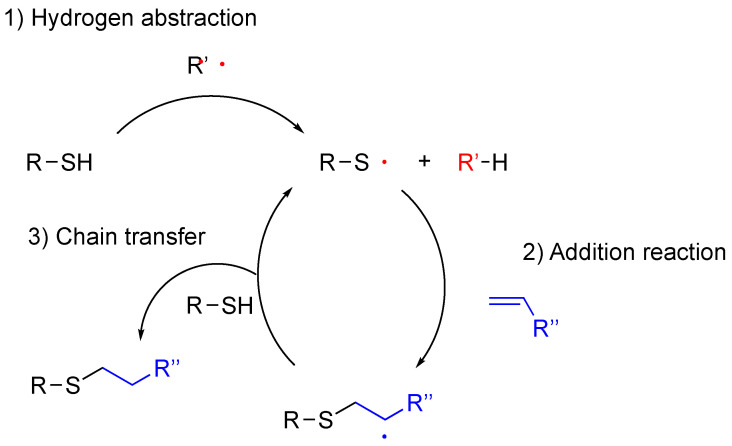
Reaction mechanism of radical hydrothiolation of alkenes consisting of hydrogen abstraction, addition to alkene, and final chain transfer step to produce thioether and another radical.

Thiol-ene reactions can be spatiotemporally controlled by introducing a radical photoinitiator activated only with irradiation. The reaction mechanism of the radical thiol-ene reaction is indicated in Figure 14. In essence, a thiol is transformed into a thiyl radical by a radical initiator with triggers of heat or light (the hydrogen abstraction process). The thiyl radical is added to an alkene to create an sp_3_ carbon-centered radical (the addition step). This radical subsequently abstracts a hydrogen atom from another thiol to produce the thioether product and regenerates a thiyl radical, which propagates the cycle again (the chain transfer step).

The radical addition proceeds faster for non-conjugated alkenes, and the low polymerizability of non-conjugated alkenes allows for selective thiol addition without polymerization of alkenes. Accordingly, non-conjugated alkenes are effective for the grafting-to method. By contrast, the thiol-ene reaction to styrene or methacrylates, which yields resonance-stabilized radical intermediates, proceeds more slowly due to the much lower hydrogen abstraction rates. As a result, chain growth leading to polymerization often accompanies this, and these alkenes fit thiol-ene reactions accompanied by propagation.

**Figure 15:**
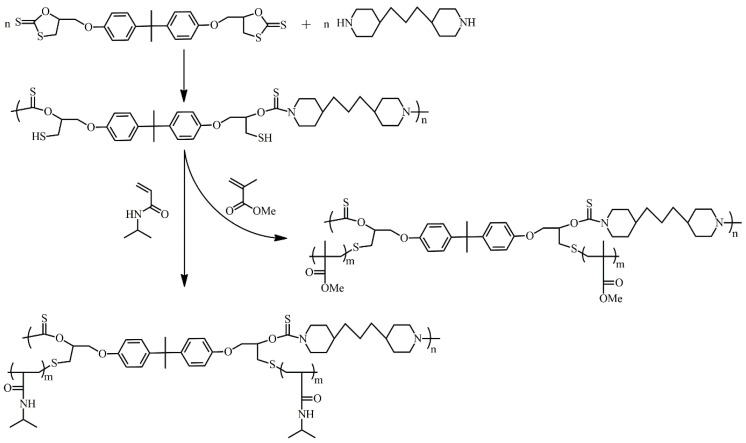
Synthesis of poly(mercaptothiourethane)-*g*-PMMA/PNIPAM by thiol-ene grafting-from polymerization from backbone bearing thiol side chains.

As an example of the thiol-ene grafting-from method, Ochiai and coworkers utilized a macro chain-transfer agent bearing thiol side chains via thiol-ene addition followed by free radical polyaddition (Figure 15) [38]. The macro-chain-transfer agent, poly(mercaptothiourethane), is a linear polymer bearing thiol moieties in the repeating unit prepared by polyaddition of a bifunctional cyclic dithiocarbonate with a diamine, which is based on a click reaction to produce thiol moieties through ring-opening of dithiocarbonates by amines. Subsequent grafting-from polymerization of MMA yielded poly(mercaptothiourethane)-*g*-PMMA through thiol-ene chemistry accompanied by propagation. The molecular weight of graft copolymers can be controlled by feed ratios of MMA to the stem chain.

This strategy was further extended to produce a thermo-responsive graft copolymer for the extraction of Pd^2+^ [39]. Grafting-from polymerization of *N*-isopropyl acrylamide (NIPAM) from poly(mercaptothiourethane) yielded poly(mercaptothiourethane)-*g*-PNIPAM, which selectively adsorbed Pd^2+^ through the thiocarbonyl group in homogeneous solutions at ambient temperature and was easily separated by precipitation at an elevated temperature. The in situ preparation of the backbone polymer prevented undesired crosslinking by oxidative coupling of thiol side chains. However, the graft polymerization based on free radical processes resulted in broad polydispersities, and monomers terminating mainly with recombination are basically unavailable to obtain soluble polymers.

**Figure 16:**
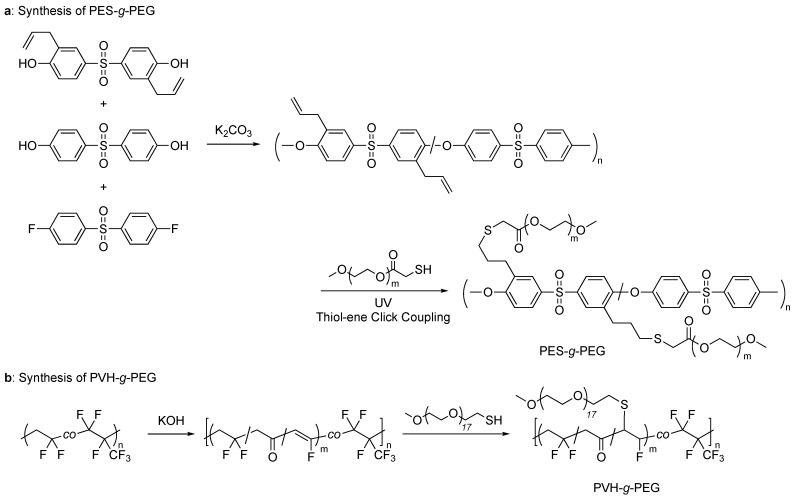
Thiol-ene grafting of PEG-SH onto (**a**) poly(ether sulfone) (PES) and (**b**) poly(vinylidene fluoride-*co*-hexafluoropropylene) (PVH) backbones with non-conjugating alkene moieties.

The grafting-to method on thiol-ene chemistry typically employs a backbone polymer bearing multiple non-conjugated alkene moieties in the side chains and a linear polymer with a thiol chain end to avoid the crosslinking of backbone polymers that often occurs for polymers with thiol side chains. Yu and coworkers utilized thiol-ene click chemistry to graft PEG onto poly(ether sulfone) (PES) [40]. PES with pendant allyl groups (PES-A) was prepared by introducing bis(3-allyl-4-hydroxyphenyl)sulfone into a polycondensation reaction of bis(4-dihydroxyphenyl)sulfone and bis(4-fluorophenyl)sulfone via the Williamson reaction. PEG-SH, separately produced by esterification of PEG with thioglycolic acid, was coupled onto PES-A via thiol-ene click chemistry to form comb-like PES-*g*-PEG. The reaction was carried out in the presence of a photoinitiator and under UV irradiation. The copolymer showed micro-phase separation between PES and PEG segments, which could be tuned by the densities and lengths of the PEG side chains. The PES-*g*-PEG membrane was successfully utilized for CO_2_ separation, with a CO_2_ permeability of 26.8 Barrer and a CO_2_/N_2_ selectivity of 27.6 (Figure 16a).

Kwon et al. also used thiol-ene chemistry to graft PEG chains onto poly(vinylidene fluoride-*co*-hexafluoropropylene) (PVH) backbone [41]. The PVH membrane prepared by the static breath-figure method was treated with KOH to generate C=C and C=O groups by dehydrofluorination. The C=C groups acted as anchoring sites for PEG-SH chains, and the PVH-*g*-PEG was obtained by the photoinitiated thiol-ene click reaction (Figure 16b). The graft copolymer was applied as a solid electrolyte of Na-ion batteries, which exhibited high electrolyte uptake, uniform Na+ distribution, and effective Na dendrite suppression.

**Figure 17:**
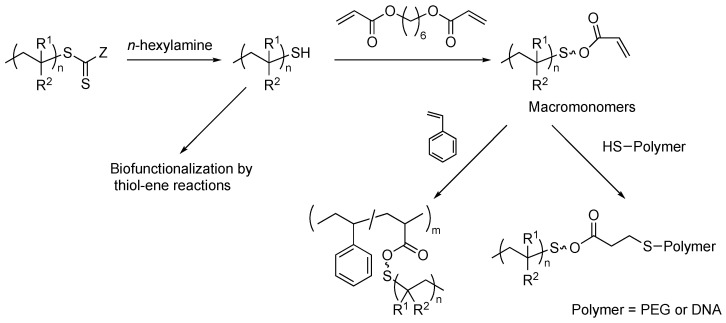
Synthesis of graft copolymers by grafting-through copolymerization of macromonomers produced by simultaneous aminolysis and thiol-ene coupling of RAFT termini polymers, and application of this strategy to the synthesis of various architectures.

RAFT termini polymers have the potential to be modified as macromonomers by virtue of thiol-ene click chemistry [23,42,43,44]. One of the challenges of modifying RAFT polymers is avoiding the formation of thiolactone [45], which loses the reactivity of end-groups. One way to prevent side reactions is in situ aminolysis and thiol-ene addition with an ene-bearing compound, such as a (meth)acrylate and a maleimide. An example was reported by Boyer and coworkers [46]. PNIPAM and PMMA were separately prepared by RAFT polymerization. Then, the sequential aminolysis and thiol-ene coupling reaction proceeded in one pot by reacting RAFT polymers with triethylamine, *n*-hexylamine, and 1,6-hexanediol diacrylate (Figure 17). The acrylate-functionalized macromonomers were copolymerized with styrene by FRP to produce PS-*g*-PNIPAM. The conversion of macromonomers was up to 75%. This strategy was applied to the synthesis of block copolymers and biofunctionalized polymers potentially applicable to biomedical applications. PEG, DNA, saccharides, and biotin were conjugated by thiol-ene click reaction.

### 3.3. Thiol-Yne Click Reaction

**Figure 18:**
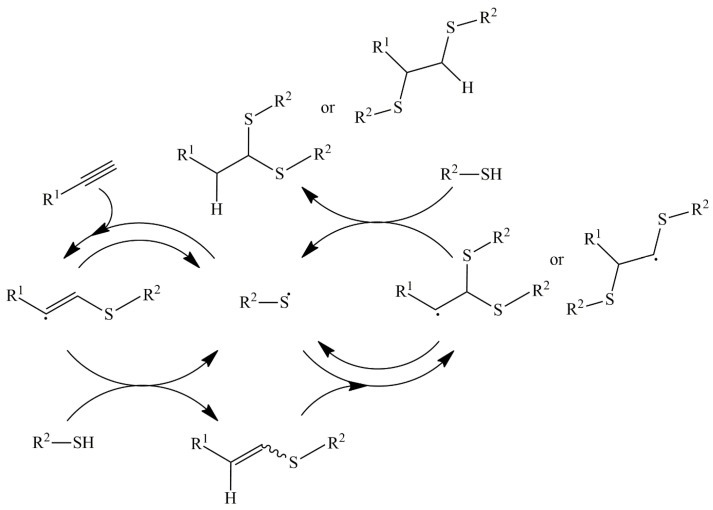
Two-step reaction mechanism of mono-addition and bis-addition of thiol to yne group by thiol-yne chemistry.

Alkynes also react with thiols, and this reaction is applicable to the synthesis of graft copolymers. Thiol-yne reactions may be preceded by radical, nucleophilic, and transition-metal-catalytic processes, but the radical process is mostly used. The initial stages for the thiol-yne reaction are the same as those for the thiol-ene reaction (Figure 18). The major differences from the thiol-ene reaction are (i) the lower reactivity of the radical intermediates to carbon–carbon multiple bonds and (ii) the possibility of dual addition. Alkenyl radicals are less stable than alkyl radicals, and their higher reactivity leads to smooth hydrogen abstraction from thiols to produce thiyl radicals. By contrast, the addition of alkenyl radicals to alkynes is not favorable. The selectivity of these single and dual additions depends on the substrates and reaction conditions, and both of the addition modes have been applied for graft copolymers [47,48]. For these factors, the chain growth of alkynes is not preferable.

For example, aryl acetylenes are effective in selective monoaddition owing to the stabilized benzylic radicals. As a result, when one equivalent of a thiol is employed in conjugation with aryl acetylene derivatives, monoadducts are selectively produced, which contrasts sharply with the thiol-yne reaction of aliphatic alkynes generally accompanied by dual additions.

**Figure 19:**
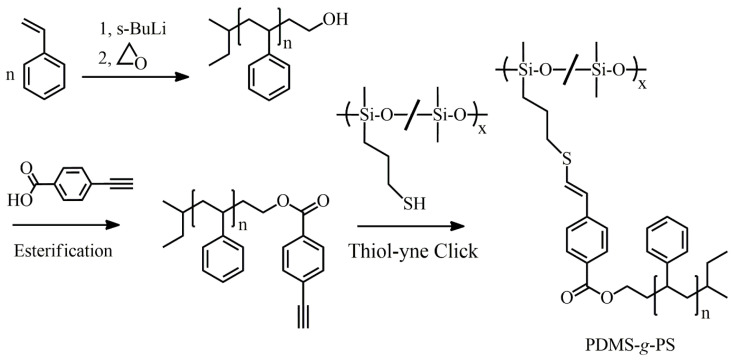
Synthesis of PDMS-*g*-PS by thiol-yne monoaddition of PS-yne, produced by sequential end capping and post-modification of living PS, as well as thiol-bearing PDMS.

Sprafke et al. applied thiol-yne monoaddition click chemistry to the synthesis of polydimethylsiloxane (PSMS) grafted by PS [49]. PS end-functionalized with an aryl alkynyl group (PS-yne) was grafted onto a commercially available PDMS stem bearing mercaptopropyl side chains (PDMS-SH). PS-yne was produced by sequential end-capping and post-modification of living PS-Li with ethylene oxide and ethynyl benzoic acid, respectively. PDMS-SH and PS-yne were coupled through the thiol-yne monoaddition in the presence of 2,2-dimethoxy-2-phenylacetophenone as a photoinitiator under UV irradiation (Figure 19). The addition proceeded completely without residual reactive groups, and the molecular weights of the resulting PDMS-*g*-PS were controlled by the lengths of living PS.

Thiol-yne monoaddition to aryl alkynyl groups is a sensitive but powerful route for the synthesis of graft copolymers. Although it requires careful optimization of reaction conditions and equimolar stoichiometry of the reactants, high overall yields, effective coupling, and functional group tolerance make it a valuable tool for synthesizing a wide variety of functionalized polymers.

**Figure 20:**
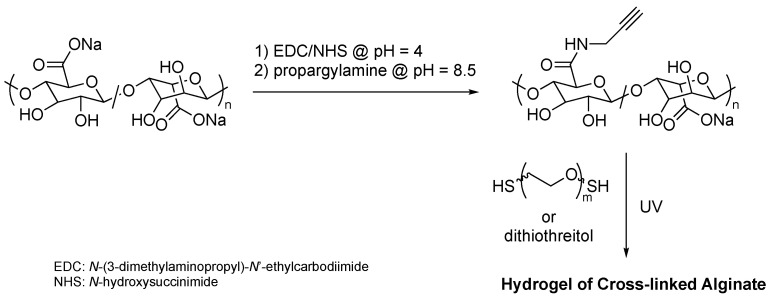
Thiol-yne cross-linking graft-onto reaction of alginate-bearing alkynyl moieties and PEG dithiol for development of biosafe hydrogels.

Hydrogels based on alginate and PEG for regenerative surgery were prepared by thiol-yne click reaction of alginate with alkynyl side chains and PEG dithiol (Figure 20) [50]. Propargyl groups were introduced to the side chain of sodium alginate from brown algae, and the resulting alkynylated alginate was cross-linked by a thol-yne reaction using PEG dithiol and 1,4-dithiothreitol. The obtained hydrogels showed sufficient mechanical strength and no cytotoxicity. The soluble extracts did not inhibit cell proliferation measured in terms of metabolic activity and DNA content. Various examples of modification of polysaccharides to graft copolymers by click chemistry were summarized in a previous excellent review [51].

## 4. Conclusions

Graft copolymers offer precise control over properties due to the discrete nature of backbone and side chains. Click chemistry achieved the formation of complex, multifunctional graft copolymers with a range of applications due to their robustness and applicability to a broader range of functional groups for monomers and polymers. CuAAC is highly efficient and specific and allows for the fabrication of a wide range of graft polymers. However, copper catalysts are often undesirable for biomedical applications due to their cytotoxicity. Researchers aiming for biomedical and biological applications focus on SPAAC as a biorthogonal alternative. Thiol-ene reactions offer high efficiency, specificity, and spatiotemporal control by UV irradiation. However, side reactions of alkenes and thiols, such as polymerization and dimerization, should be suppressed, and UV irradiation and radical sources sometimes affect other functional groups. Thiol-yne reactions have similar characteristics to thiol-ene reactions. The dual addition specific to thiol-yne reactions is an essential difference, and their control is vital to obtain desired graft polymers. The choice of click chemistry will depend on the application and properties desired for the resulting graft copolymers. In addition, combining CRPs with click reactions has emerged as a promising approach for synthesizing designed graft copolymers. The applications of graft copolymers fabricated by click reactions introduced in this review are organic electronics, batteries, medicine, scavenging of noble metals, and CO_2_ separation. Future research in this field will develop more diverse and complex materials for emerging areas, such as advanced sensors, energy-related applications, and therapeutics.

## Data Availability

All data are indicated in the references.
